# Superabsorbent poly(acrylic acid) and antioxidant poly(ester amide) hybrid hydrogel for enhanced wound healing

**DOI:** 10.1093/rb/rbaa059

**Published:** 2021-04-20

**Authors:** Jianhua Zhang, Junfei Hu, Baoshu Chen, Tianbao Zhao, Zhipeng Gu

**Affiliations:** 1 School of Materials Science and Engineering, Xihua University, Chengdu 610039, P.R. China; 2 College of Polymer Science and Engineering, State Key Laboratory of Polymer Materials Engineering, Sichuan University, Chengdu 610065, P.R. China; 3 College of Chemistry, Sichuan University, Chengdu 610065, P.R. China; 4 Yibin Tianyuan Grp Co., Ltd., Yibin, Sichuan Province 644000, P.R. China; 5 Research Institute of Sun Yat-Sen University, Shenzhen 518057, P.R. China

**Keywords:** poly (acrylic acid)/poly (ester amide), superabsorbent, antioxidant, wound healing

## Abstract

Wound healing dressing is increasingly needed in clinical owing to the large quantity of skin damage annually. Excessive reactive oxygen species (ROS) produced through internal or external environmental influences can lead to lipid peroxidation, protein denaturation, and even DNA damage, and ultimately have harmful effects on cells. Aiming to sufficiently contact with the wound microenvironment and scavenge ROS, superabsorbent poly (acrylic acid) and antioxidant poly (ester amide) (PAA/PEA) hybrid hydrogel has been developed to enhance wound healing. The physical and chemical properties of hybrid hydrogels were studied by Fourier-transform infrared (FTIR) absorption spectrum, compression, swelling, degradation, etc. Besides, the antioxidant properties of hybrid hydrogels can be investigated through the free radical scavenging experiment, and corresponding antioxidant indicators have been tested at the cellular level. Hybrid hydrogel scaffolds supported the proliferation of human umbilical vein endothelial cells and fibroblasts, as well as accelerated angiogenesis and skin regeneration in wounds. The healing properties of wounds in vivo were further assessed on mouse skin wounds. Results showed that PAA/PEA hybrid hydrogel scaffolds significantly accelerated the wound healing process through enhancing granulation formation and re-epithelialization. In summary, these superabsorbent and antioxidative hybrid hydrogels could be served as an excellent wound dressing for full-thickness wound healing.

## Introduction

Wounds caused by illness, trauma, and accidents have been a common clinical problem [1–3]. Until now, many dressings have been developed and have achieved certain results [4–6]. To the best of our knowledge, the designs of these dressings mainly focus on providing a closed microenvironment to prevent bacterial infections and promote the proliferation of relevant cells with their distinct features that benefit for wound healing. However, these strategies have some limitations that they have lost sight of the inflammatory response in the early-stage and the structural remodeling in the maturated stage [7–9]. In recent years, it has been widely acknowledged that the key to facing the challenge of these two stages is the regulation of reactive oxygen species (ROS) [10–12]. ROS in excessive inflammatory response after wound could break the localized tissue regeneration [13]. Consequently, the development of antioxidant strategy-based dressings for effective ROS scavenging has been regarded as a feasible strategy for forward enhancing wound healing [14, 15].

Advances in hydrogels have enabled several ways of ROS scavenging dressings for enhanced wound healing [16–19]. Among them, one promising way is to load antioxidants to provide the ROS scavenging ability into hydrogels. For instance, Shiekh et al. [20] have reported an oxygen-rich outer body composed of polyurethane releases antioxidant wound dressing, which can prevent infection and ulcers, and promote wound healing by increasing collagen deposition and re-epithelialization. Another way is to develop antioxidant hybrid hydrogels via grafting, coating and/or physical modification to vest them the capacity of ROS clearance. For example, Liang *et al*. [21] have prepared adhesive hemostatic antioxidation type photothermal antibacterial hydrogel based on hyaluronic acid-grafted graphene oxide and dopamine, which can promote complete skin regeneration. Besides, our previous study also has assisted arginine derivatives into the dopamine–hyaluronic acid hybrid hydrogel to enhance wound healing [22]. However, it is noting that most of these antioxidants-strategies-based dressing focus on the antioxidant capacity of the dressings and/or the controlled release of antioxidants from the dressings. The antioxidant effect mainly occurs at the wound/dressing interface lead to inadequate interactions. It remains a challenge to develop a hydrogel that has excellent ROS scavenging ability and could interact with wound tissues sufficiently.

Herein, this project intends to start from the perspective of hyper-osmotic fluid, so that the dressing can absorb excessive ROS in the fluid into the dressing. Moreover, the external dressing in contact with the wound could better achieve the removal of ROS at the wound site, thereby promoting wound healing. Therefore, this project designed a multi-functional hydrogel, which formed a new hybrid hydrogel with the superabsorbent poly (acrylic acid) (PAA) and antioxidant poly (ester amide) (PEA) via the crosslinked-polymerization of acrylic acid and the photo-polymerization of arginine-based unsaturated PEA (Supplementary Scheme S1 and S2). PAA hydrogel has been well known as a high water-absorbing and protein resistive hydrogel material used in biomedical fields. Arginine-based unsaturated PEA with amide blocks, ester blocks and arginine residues in the chain has been demonstrated excellent biocompatibility and biodegradability, which could maintain and perform the function of arginine after biodegradation. The PAA/PEA hybrid hydrogel combines the concepts of high hygroscopicity and antioxidant, which make the hydrogels absorb and interact with the exudate to perform ROS scavenging. This study provides an attractive way to develop effective ROS scavenging hydrogels which could serve as a promising strategy for wound healing.

## Experimental

### Materials

Chemical reagents for material synthesis and hydrogel preparation include L-arginine, *p*-toluenesulfonic acid monohydrate, ethylene glycol, *N*, *N*’-methylene bisacrylamide (MBA), (NH_4_)_2_S_2_O_8_, *N*, *N*, *N*’, *N*’-tetramethylethylenediamine (TMEDA), toluene, isopropanol and triethylamine were provided by Aladdin. NIH 3T3 cells were obtained from Sun Yat-sen University. Cell experiment related reagents include penicillin-streptomycin, 0.25% trypsin-EDTA, fetal bovine serum (FBS) and Dulbecco’s Modified Eagle Medium (DMEM) were purchased from Invitrogen, 3-(4,5-dimethyl-2-thiazolyl)-2,5-diphenyl-2H-tetrazolium bromide (MTT), fluorescent dyes include Calcein-AM, propidium iodide (PI), TRITC Phalloidin and Hoechst were provided by Sigma-Aldrich.

### Fabrication of PAA/PEA hybrid hydrogels

Arginine-based PEA has been synthesized according to a similar procedure of our previous studies [23–25], the specific methods and nuclear magnetic resonance (^1^H NMR) results (Supplementary Fig. S1) were shown in the Supplementary Information. The specific methods about the synthesis of PAA can be obtained in the Supplementary Information. The PAA/PEA hybrid hydrogels were fabricated as follows: With PEA dissolved in deionized water, 6.985 mL acrylic acid was dissolved thoroughly and defoamed by ultrasonication. Then (NH_4_)_2_S_2_O_8_, MBA and TMEDA were successively dispersed into the solution. The hydrogels were crosslinked on the polytetrafluoroethylene (PTFE) mold under 80°C humid conditions for 12 h and then put in a UV curing box for 3 min, and finally left stable in a humid environment at room temperature for 12 h. For a better demonstration of the effect of PEA in hybrid hydrogels, four PAA/PEA hybrid hydrogels with specific cylinder were prepared with different PEA contents (0%, 10%, 30% and 50% wt%) which were named as PAA, PAA/PEA 10%, PAA/PEA 30% and PAA/PEA 50%. The PAA/PEA hybrid hydrogels were stored at 4°C under vacuum preservation before characterization.

### Characterization of PAA/PEA hybrid hydrogels

Thermogravimetric analysis (TGA, Seiko Exstar 6300) and Fourier-transform infrared spectroscopy (FT-IR, SHIMADZU IRAffinity-1, Japan) with standard methods were carried out to confirm the hybrid hydrogels. Scanning electron microscope (SEM, Q25, FEI) was performed to investigate the interior morphology of hydrogels. Before observation, the mixed hydrogel was freeze-dried, extracted and placed on the sample stage for gold coating. As a superabsorbent hydrogel, the swelling kinetics of hydrogels were measured for 7 days at room temperature and permeated in 5 ml phosphate buffer saline (PBS) for a pre-determined time. The hydrogel swelling ratio (R) was calculated by the equation: *R* = [(*W*_a_−*W*_b_)/*W*_b_]×100%. *W*_a_ and *W*_b_ are the weight of the corresponding wet hydrogel at time *t* and the initially formed wet hydrogel, respectively. The water vapor transmission rate (WVTR) was measured through a gravimetric method according to ASTM E96–95 standard. The WVTR values were evaluated based on the slope of weight change and time. The WVTR was counted using the following formula: WVTR = (*W*_d_−*W*_t_)/*A*. WVTR is shown in g/m^2^h, *A* represents the area of glass cup mouth (m^2^), *W*_d_−*W*_t_ represents changes in weight. The mechanical properties of the hydrogels were evaluated in a ‘controlled force’ mode via employing Dynamic Mechanical Analyzer (DMA, CMT-200). The compression elastic modulus (*E*) of hydrogels was determined by plotting the compressive stress versus strain. Finally, *in vitro* biodegradation of PAA/PEA hydrogels was measured by testing the weight loss at the pre-determined period. Besides, arginine contents in the degradation fluid of hydrogels were determined. Determination of arginine by UV absorption spectroscopy (Tecan, Infinite M200 PRO) via a mixture of α-naphthol (methyl naphthol) and diacetyl as a color developer.

### Antioxidant capacity of PAA/PEA hybrid hydrogels

2, 2-diphenyl-1-picryl-hydrazyl-hydrate (DPPH) assay has been used to initially demonstrate the antioxidant capacity of PAA/PEA hybrid hydrogels. Briefly, a fresh DPPH/ethanol (40 mM) solution was used for the measurements. Afterward, different weight of hydrogels was permeated in 5 mL of DPPH solution for pre-determined time. The absorbance change at 517 nm was calculated using a multi-function microplate reader. DPPH radical scavenging activity was counted as the following formula: DPPH scavenging activity = (*A*_k_−*A*_s_)/*A*_k_×100%, where *A*_k_ is the absorbance of DPPH solution, *A*_s_ is the absorbance of samples mixed with DPPH solution.

### Cytocompatibility of PAA/PEA hybrid hydrogels

To investigate the cytocompatibility of PAA/PEA hybrid hydrogels, NIH 3T3 fibroblast cells purchased from Guanghua Stomatology Hospital affiliated of Sun Yat-sen University were chosen as cell lines. Concretely, NIH 3T3 cells were cultured with complete medium (containing 10% FBS and 1% antibody) and incubated in a 37°C 5% CO_2_ incubator. MTT assay was performed to demonstrate the cell viability after co-cultured with each sample. NIH 3T3 cells were cultured in 96-well plates and then the leaching solution of PAA/PEA hybrid hydrogels was added. After 3- and 5-days incubation, 10 μL of MTT solution (5 mg/mL) was added to each well and 5 h later 200 μL of dimethyl sulfoxide (DMSO) was added with shaking. The optical density (OD) value determined at 490 nm was applied to calculate the cell relative growth rate by using a Bio-TEK automatic microtiter plate reader. Live/Dead and TRITC/Hoechst staining were supplementary investigated to validate the NIH 3T3 cell viability and morphology cultured on PAA/PEA hybrid hydrogels. After 3- and 5-days treatment and incubation, the Live/Dead and TRITC/Hoechst staining were carried out based on the manufacturer’s protocol and the cell state on the PAA/PEA hybrid hydrogels were recorded using an inverted fluorescence microscope (Zeiss Axio Observer Z1, Germany). 

### Intracellular antioxidant capacity of PAA/PEA hybrid hydrogels

As an ideal antioxidant biomaterial for wound healing, the hybrid hydrogel ought to not only provide a friendly microenvironment for cell growth but also scavenge the radical of oxidized stress, which is a positive signal during wound healing. Consequently, the intracellular antioxidant capacity of PAA/PEA hybrid hydrogels was inspected to detect the generation of reactive oxygen by 2′,7′-dichlorofluorescein diacetate (DCFH-DA). The detailed steps can be obtained from Supplementary Information. Further, the molecular mechanism of the protection provided by the hydrogels was clarified by investigating the expression of several cellular oxidative stress indicators, including total glutathione to oxidative glutathione ratio (GSH/GSSG), superoxide dismutase (SOD), malondialdehyde (MDA) following their respective protocols by using detection kits; the specific methods can be obtained in the Supplementary Information.

### *In vitro* angiogenesis assay

To the best of our knowledge, well-controlled oxidative stress could be beneficial for angiogenesis during tissue regeneration. Thus, angiogenesis was analyzed using endothelial tube formation assay following the manufacturer’s instructions in our study. Human umbilical vein endothelial cells (HUVECs) were sown in a Matrigel-coated plate (96-well) at a density of 1 × 10^4^ per well. Test groups were treated with hydrogel extract. The formation of tube was evaluated at 2, 4, 6 and 8 h after seeding using a phase-contrast microscope. Besides, RT-qPCR was executed to assess the mRNA expression of osteogenic genes.

### *In vivo* animal studies

As we know, hemostatic is the first phase of healing after the injury to stop the bleeding. Thus, the hemostatic properties of PAA/PEA hybrid hydrogels *in vivo* were determined before wound healing by using the liver lobes of rats (25 ± 5 g) as models. All animals were acquired from the Institution animal center of Sun Yat-sen University and treated according to guidelines approved by the Sun Yat-Sen University Ethics Committee. For liver hemostasis, a little hole was made in the liver using 1 mL syringe. Then, the hydrogel was applied to the pinhole and gently pressed. In the process of the surgery, the blood was carefully collected with filter papers at the time point of 1 min. The total amount of blood loss was determined by weighing the papers and recorded. In wound healing model, the dorsum hair of SD rats (200 ± 20 g) was shaved and the skin surface was disinfected with 75% (vol/vol) ethanol. Subsequently, two symmetrical 12 mm full-thickness excision wounds on the back skin were cut out with aseptic surgical scissors. Randomly divided into five groups, including control, PAA, PAA/PEA 10%, PAA/PEA 30% and PAA/PEA 50% (*n* = 3 in each group). Finally, each wound was covered with Tegaderm film (3 M, St Paul, MN). All dressings were changed every three days. A camera was used to take pictures of the wound on 0, 5, 10 and 15 days after the operation. Image analysis software (NIH ImageJ) was used to quantify wound area. The mice were euthanized on 5th, 10th and 15th day after the operation, and tissues were collected for the following analysis. Skin sections were stained with Masson’s trichrome and hematoxylin and eosin (H&E) for the assessment of granulation tissue formation and wound maturity. Moreover, the sections were incubated with rat anti-CD31 and anti-VEGF antibody for immunohistochemical staining, as well as IL-6 and TNF-α, were also evaluated to demonstrate the inflammatory response. Moreover, an infectious wound model was performed to evaluate the hydrogels. The details are shown in the Supplementary Information.

### Statistics analysis

Data are expressed as calculated on the average at least three data points mean ± standard error. Compared with the control group, the significant difference was assessed by the unpaired Student’s *t*-test (*P* < 0.05). Data analysis was performed using Origin software.

## Results and discussion

### Fabrication and characterization of PAA/PEA hybrid hydrogels

**Figure 1.**
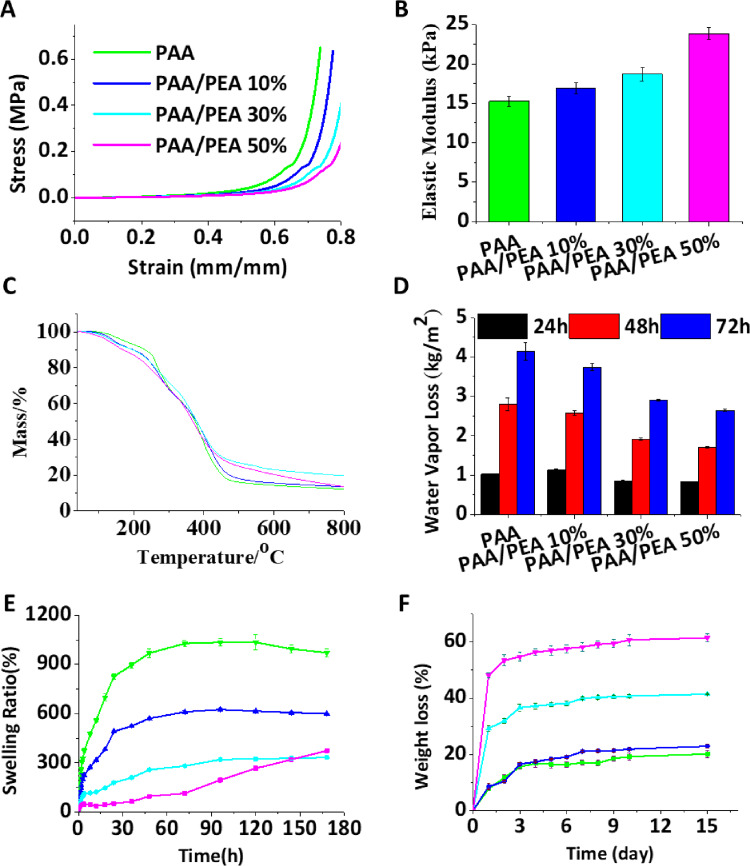
Characterization of hydrogels. (**A**) Stress-strain profiles of hydrogels by compression; (**B**) Elastic modulus; (**C**) TG analysis; (**D**) Transport properties of hydrogels; (**E**) Swelling ratios of hydrogels in PBS (pH 7.4) at 37°C; (**F**) Degradation profiles of the hydrogels in PBS with pH 7.4 at 37°C.

As shown in Supplementary Figs. S2 and S3, the fabricated PAA/PEA hybrid hydrogels seem like see-through gummy candies and turned to a yellow tint with the PEA addition. Compared to the PAA hydrogels, PAA/PEA hybrid hydrogels became fragile and showed the porous architecture in the internal morphology. To the best of our knowledge, suitable mechanical properties of hydrogels could be good for wound healing which can not only maintain the integrity of the hydrogels but also beneficial to fit the skin tissue deformed by the external force [26]. Figure 1A and B exhibited the compression stress–strain measurements that the increased mass ratio of PEA in the hydrogels showed lower stress and higher elastic modulus at the same strain. This phenomenon might be due to the hydrogen bonding network of PAA broken by the incorporation of PEA. The rheological properties of PAA/PEA hydrogels (Supplementary Fig. S4) showed a lower G′ of PAA/PEA hybrid hydrogels than the PAA group which could verify the assumption. Besides, TG analysis (Fig. 1C), water vapor loss (Fig. 1D) and swelling behavior (Fig. 1E) were also demonstrated to evaluate the influence of PEA for PAA/PEA hydrogels. The water vapor loss of PAA hydrogel is highest and decreases with the addition of PEA. The swelling behavior of hydrogels further affirmed the possibility of absorbing wound exudate and reducing the probability of infection. All of the hydrogels reached a maximum water absorption ∼300% of their initial weight after 7 days. The highest water absorption of about 1034% was observed for PAA hydrogel over 7 days, and 625%, 333% and 320% for PAA/PEA 10%, PAA/PEA 30% and PAA/PEA 50%, respectively. From all the above results, we could conclude that the decline of hydrogels’ water maintaining capacity is mainly because the addition of PEA leads to the destruction of hydrogen bonds. Therefore, the content of PEA within the proper range in the PAA/PEA hydrogels can only be regarded as the suitable hydrogel for wound healing. Moreover, a suitable degradation rate is also one focal issue for the optimization of biomaterials to match the wound healing process. Figure 1F represents the degradation study results of PAA/PEA hybrid hydrogels within 15 days. The degradation rate of PAA hydrogel was about 20%, while it rose from 22% to 60% with the increase of PEA. These data demonstrated that the biodegradation of PEA and the hydrogen bonding network broken by the incorporation of PEA are the main contributors of degradation rate changing for PAA/PEA hybrid hydrogels. In summary, all the results indicate that the PAA/PEA hybrid hydrogel with proper microstructure, super hygroscopicity and satisfactory degradation performance will have good wound healing potential.

### Arginine release and antioxidant abilities of PAA/PEA hybrid hydrogels

**Figure 2.**
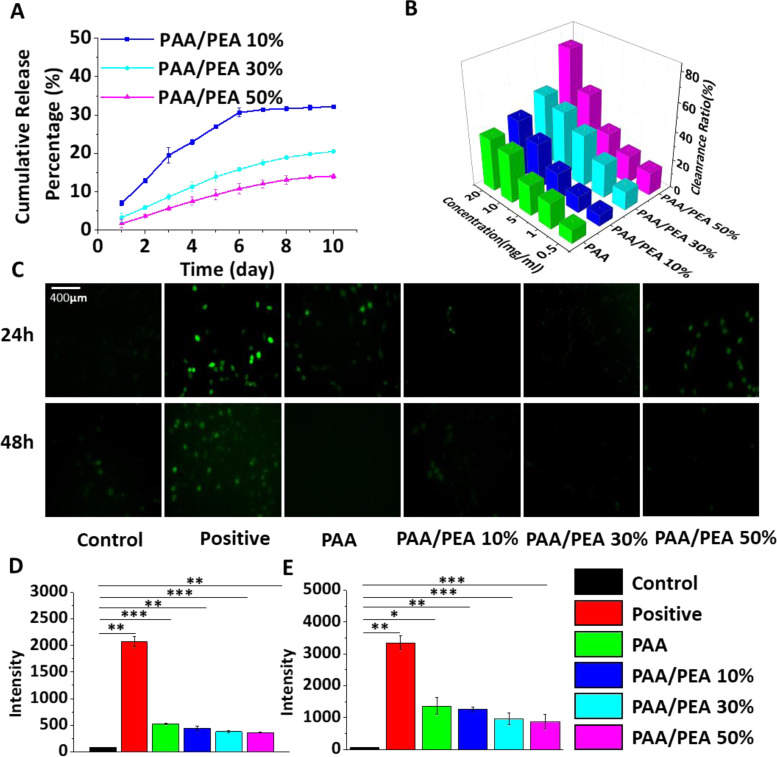
(**A**) Real time content of arginine in PAA/PEA hybrid hydrogels. (**B**) DPPH scavenging percentage by hydrogels with different concentrations at 24 h. Fluorescence images (**C**) of ROS staining by the fluorescent probe DCFH-DA in NIH 3T3 cells after 24 h (**D**) and 48 h (**E**).

In PAA/PEA hybrid hydrogels, arginine is the main contributor of ROS scavenging ability. Thus, arginine release has been tested to initially demonstrate the scavenging time for wound healing. As shown in Fig. 2A and Supplementary Fig. S5, it can be concluded that the release of arginine from PAA/PEA hybrid hydrogels could maintain for more than 7 days in each group, which could perform their ROS scavenging capacity during the inflammatory process of wound healing. More specifically, PAA/PEA 50% release the most arginine during the degradation, while the release rate is the lowest. The typical free radical scavenging ability of PAA/PEA hydrogels was estimated through their reaction with stable DPPH radicals by using the previous methodology (Fig. 2B). All PAA/PEA groups exhibited good ROS scavenging ability and this ability depends on the usage of hydrogels and the content of arginine in the hydrogels. Further, the intracellular antioxidant behaviors of PAA/PEA hybrid hydrogels for the protection of NIH 3T3 cells against the oxidative stress were examined. Before this, a cytocompatibility testing was performed to make sure that the PAA/PEA hybrid hydrogels at the state could do no harm to cell proliferation and spread (Supplementary Fig. S6). Then cells incubated with hydrogels were treated with H_2_O_2_ to investigate the antioxidant ability at the cellular level. As shown in Fig. 2C–E by DCFH-DA stain and flow cytometry, cells would generate more intracellular ROS after treated with H_2_O_2_, while the ROS level could be declined when cultured with PAA/PEA hybrid hydrogels depending on the PEA ratio. These results indicated that PAA/PEA hybrid hydrogels have excellent ROS scavenging ability at the intracellular level mainly according to the contribution of the release of arginine.

**Figure 3.**
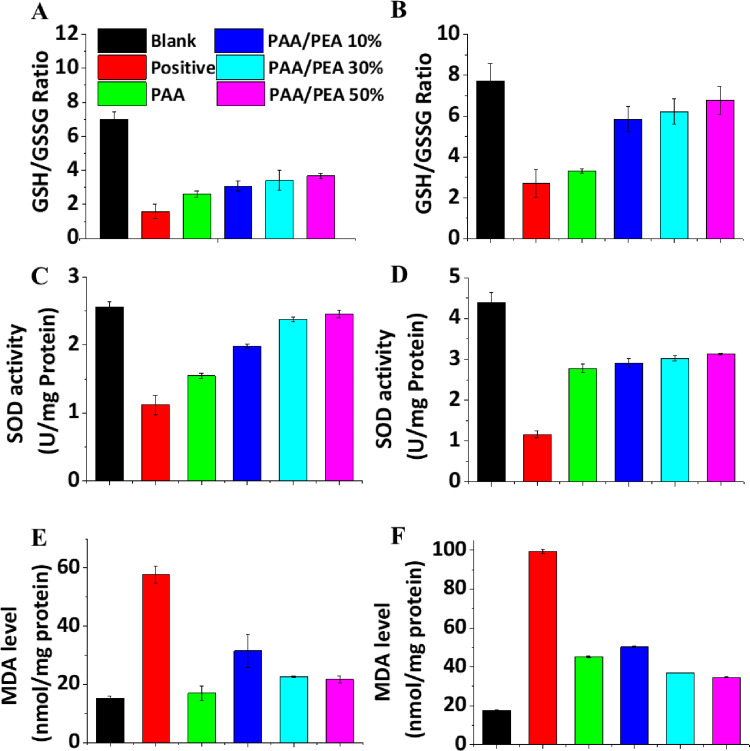
PEA inhibited the changes of GSH/GSSG, SOD and MDA in a concentration-dependent manner. After incubation for 24 and 48 h with different compounds as indicated, cells were harvested, and the contents of GSH/GSSG (**A** and **B**), SOD (**C** and **D**) and MDA (**E** and **F**) were determined, respectively.

To further investigate the mechanism of PAA/PEA hybrid hydrogels inhibiting ROS-induced cell apoptosis, the status of GSH/GSSG, SOD, and MDA of H_2_O_2_-treated cells incubated with/without hydrogels extracted at 24 h and 48 h has been examined (Fig. 3). To the best of our knowledge, when oxidative stress becomes prolonged and cellular systems are no more able to counteract the abundance of oxidative stress, the ratio of GSH/GSSG and SOD level will reduce. MDA is one of the final products of polyunsaturated fatty acids peroxidation in the cells which could be induced by free radicals. The results from Fig. 3 demonstrated the antioxidant of PAA/PEA hybrid hydrogels might influence the multi-pathway of cells self-protected against oxidative stress. Moreover, angiogenesis is the formation of new blood vessels which is also a key factor for wound healing. For now, various progresses have been made to examine the angiogenesis promotion of biomaterials. A promising way is to translate biomaterials over to living organisms; however, the operation is hard to implement so that several investigations have built to demonstrate that synthetic antioxidants might reduce or inhibit angiogenesis in some cases [27–29]. To evaluate the angiogenesis ability of cells cultured with PAA/PEA hybrid hydrogels, the number of tubes formed by HUVECs was tested after incubating the cells with the hydrogel extract for 2 and 4 h (Supplementary Fig. S7). Besides, CD31 as an endothelial cell receptor of angiogenesis has been quantified using PCR analysis (Supplementary Fig. S8). These results suggested that the antioxidant ability from arginine also can promote early angiogenesis which benefits the rapid formation of granulation tissue.

### Evaluation of PAA/PEA hybrid hydrogels *in vivo*

**Figure 4.**
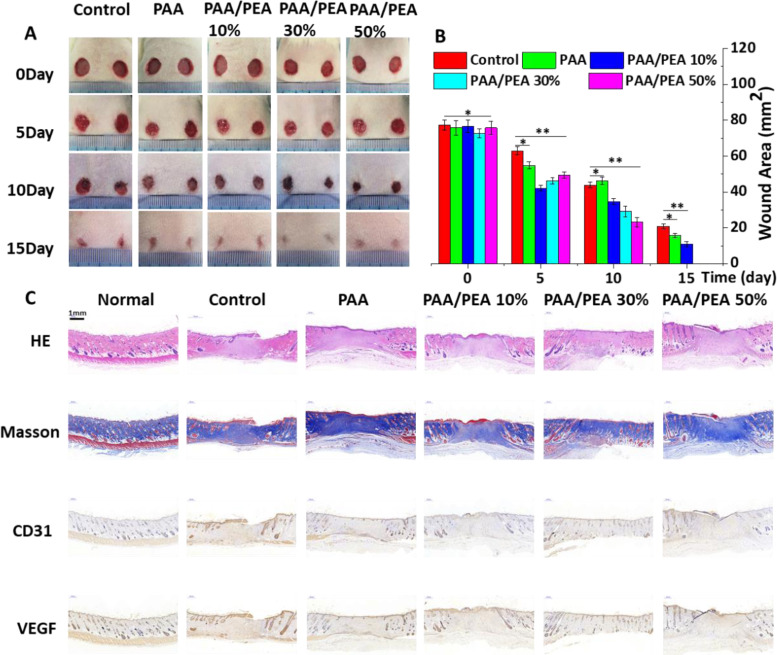
(**A**) Representative images of wounds treated with PAA, PAA/PEA10%, PAA/PEA 30% and PAA/PEA 50% hydrogels. (**B**) Wound repair area of different experimental groups. Representative histological H&E, Masson, CD31 and VEGF stained sections of wounds of rats treated on day 15 (**C**).

Before the *in vivo* full-thick wound study, the hemostatic activities of PAA/PEA hybrid hydrogels were performed. The results showed that all of the PAA/PEA hybrid hydrogels could stop the bleeding of a liver hole section in several seconds. In particular, PAA/PEA hydrogels sealing had more prominent hemostasis properties than the untreated controls and PAA hydrogel treatment groups (Supplementary Fig. S9). These results initially demonstrated the good wound sealing and halt blood loss capacities of PAA/PEA hybrid hydrogels that could be beneficial for wound healing. Moreover, the representative images of wounds treated with various hydrogels in the process of wound healing have been shown in Fig. 4A and their wound area was quantified in Fig. 4B. It can be seen that compared with the PAA single hydrogel and control, all those wounds treated with PAA/PEA hybrid hydrogels achieved a larger reduction in wound area. And all those wounds treated with PAA/PEA hybrid hydrogels can also reduce the risk of wound infection to a certain extent (Supplementary Fig. S10). What’s more, such a wound area reduction becomes apparent as early as day 5. The wound healing rates obviously sped up by PAA/PEA 10%, PAA/PEA 30% and PAA/PEA 50%. The PAA/PEA 50% group brought about the highest healing rate, followed by PAA/PEA 30%, PAA/PEA 10%, PAA and control on day 10. On the 15th day, the wounds’ healing rate in the PAA/PEA 50% and PAA/PEA 30% groups expressed more than 95%, illustrating that the design concept of superabsorbent PAA and antioxidant arginine-based PEA hybrid hydrogel significantly accelerated the wound healing process of rat models. It is worth noting that the wounds’ healing effect in the PAA/PEA 30% groups was the most significant. The epidermis has been well repaired, the size of the wound is basically zero, and the structural remodeling of the dermis is relatively perfect, its depth is no different from that of the surrounding normal tissue, and the repaired skin has a smooth appearance without wrinkles, which has a good potential for commercial transformation. What’s more, the hydrogel material can also regulate the immune microenvironment to a certain extent, reduce the expression of IL-6 and TNF-α (Supplementary Fig. S11), thereby speeding up wound healing. In addition, we have further demonstrated the effect of hydrogel dressings through infection models (Supplementary Fig. S12). More importantly, the results of H&E staining, Masson staining, CD 31 and VEGF immunohistochemical staining in Fig. 4C showed that the addition of PEA might be beneficial for the structural remodeling stage via the observation of collagen fibers structure, deposition and hair follicle structure, etc.

## Conclusion

In summary, we present a series of superabsorbent PAA and antioxidant arginine-based PEA hybrid hydrogels and verified their performance in cell and rat models. These PAA/PEA hybrid hydrogels were successfully prepared and demonstrated that exhibited multi-function like superabsorbent swelling, suitable degradation behavior and excellent biocompatibility. Additionally, antioxidant property and hemostatic effects were also indicated by the free radical scavenging test, cell culture and mouse liver injury model. Furthermore, in the full-thickness mouse skin wound model, histomorphology evaluation and immunohistochemical staining during wound healing showed that PAA/PEA hybrid hydrogels can significantly accelerate the wound healing process and effectively improve the tissue structure of regenerated tissue. All these results demonstrated that superabsorbent PAA and antioxidant arginine-based PEA hybrid hydrogels with multiple functions might be ideal candidates as wound healing dressing.

## Supplementary data

Supplementary data are available at *REGBIO* online.

*Conflict of interest statement.* The authors declare no conflict of interest. 

## Supplementary Material

rbaa059_Supplementary_Data
