# Supplementary material for: Superabsorbent poly(acrylic acid) and antioxidant poly(ester amide) hybrid hydrogel for enhanced wound healing
Source: Regen Biomater. 2021 Apr 20;8(2):rbaa059. doi: 10.1093/rb/rbaa059 (PMC8055781; doi:10.1093/rb/rbaa059)
Supplement: rbaa059_Supplementary_Data [file rbaa059_supplementary_data.docx]

**Supporting Information**

**Superabsorbent** [**polyacrylic**](javascript:;) [**acid**](javascript:;) **and antioxidant poly(ester amide) hybrid hydrogel for enhanced wound healing**

Jianhua Zhang^1, 2^, Junfei Hu^2^, Baoshu Chen^1^, Tianbao Zhao^1,4,5^*, Zhipeng Gu^2, 3^*

*^1^* *School of Materials Science and Engineering, Xihua University, Chengdu 610039, PR. China*

*^2^ College of Polymer Science and Engineering, State Key Laboratory of Polymer Materials Engineering, Sichuan University, Chengdu 610065, PR. China*

*^3^ Research Institute of Sun Yat-Sen University in Shenzhen, Shenzhen, 518057, PR China*

*^4^ College of Chemistry, Sichuan University, Chengdu 610065, PR. China*

*^5^ Yibin Tianyuan Grp Co.,Ltd. Yibin 644000,Sichuan Province, PR. China*

** E-mail: guzhipeng2019@scu.edu.cn (Z.Gu), zhaotb@mail.xhu.edu.cn (T.Zhao)*

**Synthesis of Arg-UPEA**

In this report, L-arginine (L-Arg) were chosen as a precursor component due to their excellent biocompatibility, water solubility, low toxicity and low immunogenicity. An unsaturated di-p-nitrophenyl ester of dicarboxylic acid (Monomer Ⅰ, di-p-nitrophenyl fumarate (NF), x=2) was prepared by reacting fumaryl chloride with p-nitrophenol. One type of p-toluenesulfonic acid salts of L-arginine diester (Monomer Ⅱ) was prepared in this study: the tetra-p-toluenesulfonic acid salt of bis(L-arginine) ethane diester Arg-2-S (y=2). Arg-UPEA was prepared by the solution polycondensation of (Ⅰ) and (Ⅱ) monomers (NF and Arg-2-S). The synthesis scheme of the monomers and polymer could be found in the Scheme S1. Arg-UPEA synthesize with combination of diacid and diol building blocks were: 2-UArg-2-S. The structure of the Arg-UPEA was confirmed by ^1^H NMR. The 1H NMR peaks marked with numbers from 1 to 11 are assigned to the corresponding protons of 2-UArg-2-S as shown in Figure S1. The results of the three tests are basically consistent, indicating that the synthesis and purification methods are correct, and the products can be stably produced.

**Synthesis of PAA**

Acrylic acid monomer is easy to polymerize, and solution polymerization is used in this experiment, which accords with the general law of free radical polymerization. In order to facilitate the preparation of hydrogels, PEA was added in advance in the process of PAA polymerization, and PAA/PEA hybrid hydrogels could be obtained after the end of the reaction. With PEA dissolved in deionized water, 6.985 mL acrylic acid was dissolved thoroughly and defoam the ultrasonication. Then (NH_4_)_2_S_2_O_8_ (0.0733 g), MBA and TMEDA (0.5 wt% to acrylic acid) were successively dispersed into the solution. Finally, the reaction solution was placed in a hot and humid environment of 80 ^o^C for 12 hours.

**Intracellular antioxidant capacity of PAA/PEA hybrid hydrogels**

NIH-3T3 cells were first seeded in 6-well plates and cultured overnight. Afterward, the adherent cells were incubated with hydrogel extract (the concentration of extract is 5 mg/mL) for 12 h under 37 °C. In order to remove the excess hydrogel extract, cells were washed three times with PBS. The cells were then incubated at 37 °C for 24 h and 48 h with 9.8 µM H2O2. Then, the cells were further incubated with DCFH-DA at 37 °C for 30 min. After being washed three times with PBS, intracellular ROS levels were measured with the inverted fluorescence microscope and flow cytometer.

***In vitro* antioxidant ability of hydrogels**

Total glutathione (GSH) and oxidative glutathione (GSSG) levels were measured by the colorimetric microplate assay kits supplied by Beyotime Institute of Biotechnology, Zhejiang, China. Briefly, the cells were washed once with PBS and collected by centrifugation, and the supernatant was aspirated. Add three folds amount of protein removal reagent M solution to the cell pellet volume, then fully vortex. The sample was then subjected to two rapid freeze-thaw cycles using liquid nitrogen and a 37^o^C water bath. Then leave in 4^o^C or ice bath for 5 minutes. Centrifuge at 10, 000 g for 10 minutes at 4^o^C. The supernatant was taken for the determination of total glutathione. The total GSH level was measured by the method of DTNB-GSSG recycling assay. The GSSG level was quantified by the same method of total GSH assay after the supernatant was pretreated with 1% 1 mol/L 2-vinylpyridine solution to remove the reduced GSH. The amount of reduced GSH was obtained by subtracting the amount of GSSG from that of the total GSH.

Total superoxide dismutase (SOD) activity was measured using Total Superoxide Dismutase Assay Kit with WST-8 (Beyotime Institute of Biotechnology). The cells were collected by centrifugation at 600 g for 5 minutes, then washed with PBS or saline pre-cooled at 4 °C or ice bath, added SOD sample preparation solution at a ratio of 200 μL per 1 million cells, and appropriately blow to fully cleavage the cells. Centrifuge at 12, 000 g for 5 minutes at 4 °C, and take the supernatant as a sample to be tested. The absorbance was read at 450 nm after 30-min incubation at 37 °C. Meanwhile, the reading of blank 1 (buffer + WST-8 working solution + start solution), blank 2 (buffer + WST-8 working solution) and blank 3 (sample + buffer + WST-8 working solution) was determined and used to calculate sample results according to manufacturer’s instructions. The results of SOD activity were expressed as U/mg protein.

The malondialdehyde (MDA) levels in the NIH-3T3 cells were determined by measuring the production of thiobarbituric acid-reactive components by using the MDA assay kit (Beyotime Institute of Biotechnology, Beijing, China). Cells were lysed using Western and IP cell lysates, 0.1 mL lysate was used per 1 million cells. After lysis, the cells were centrifuged at 12, 000 g for 10 minutes, and the supernatant was taken for subsequent measurement. The results were normalized to the total protein levels, as measured by BCA protein concentration determination kit, and expressed as nmol/mg protein.

***In vitro* antibacterial ability of PAA/PEA hybrid hydrogels**

For the preculture process, Gram-positive bioluminescent S. aureus Xen36 was cultured on tryptone soy agar plates containing kanamycin at a final concentration of 200 μg/mL. Then, a single colony was transferred into 10 mL of tryptone soy broth (TSB) and incubated at 37 °C for 24 h, followed by 20-fold dilution with TSB (190 mL). After incubation for 16 h, the bacterial suspension was centrifuged (5000 g, 5 min), washed three times with PBS, sonicated (3 times × 10 s) while cooling in an ice bath to break possible aggregates, and finally dispersed in 10 mL of PBS for the subsequent experiments. Add 100 μL of the bacterial solution to the pre-treated pigskin, and then apply the hydrogel material on the pigskin. After incubating for 4 days at 37°C, fix it with 4% paraformaldehyde solution, and use gradient dehydration with different concentrations (30%, 50%, 70%, 80%, 90%, 95%, 100%) of ethanol aqueous solution, dry at room temperature, observe the sample with scanning electron microscope after spraying gold.

***Infected wound model***

Female Sprague–Dawley rats weighing ≈200 g were anesthetized with chloral hydrate with a dose of 30 mg/100 g. The dorsum skin was shaved and disinfected. Circular full-thickness wounds with a diameter of 6 mm were excised from the dorsum of each rat by a stainless-steel puncture and the skin was removed. To mimic the biofluid around the wounds, the simulated biofluid was prepared by adding bacteria (10^6^ CFU mL^−1^ S. aureus) into FBS and Maximal Recovery Diluent (MRD) at a 50/50 v/v ratio concentration, and 50 µL of simulated biological fluid was dropped on wound bed. The prepared hydrogel dressing was used to cover the wound after 24h, and the wound healing in the later period was observed.


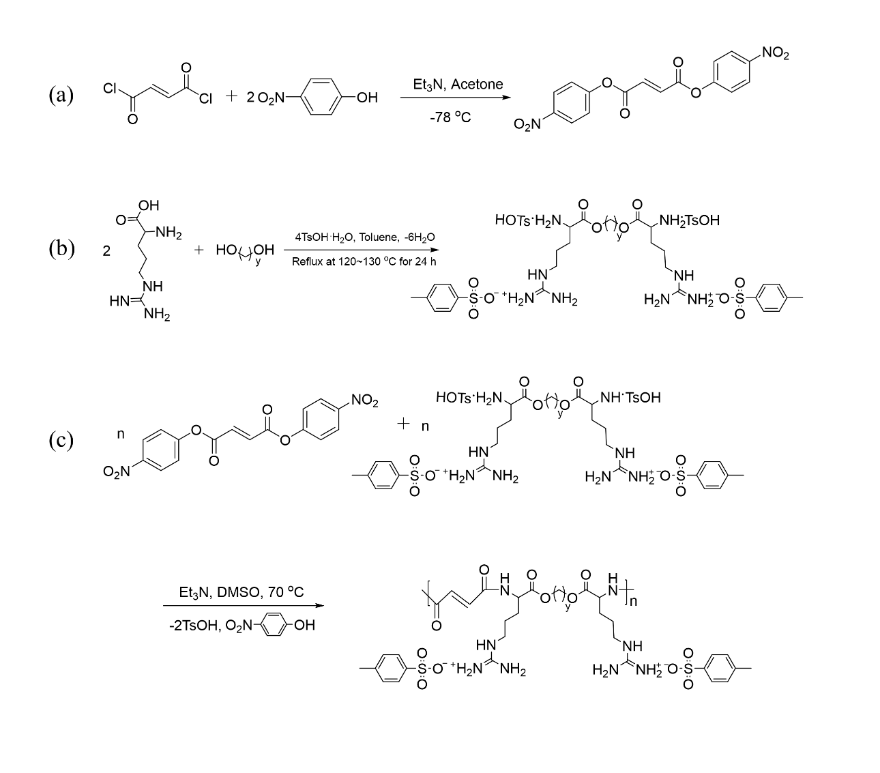


Scheme S1. The synthesis scheme of the monomers and polymer. (a) The synthesis scheme of NF. (b) The synthesis scheme of Arg-2-S, TsOH•H_2_O = p-Toluenesulfonic acid monohydrate; TsOH = p-Toluenesulfonic acid. (c) The synthesis scheme of Arg-UPEA.


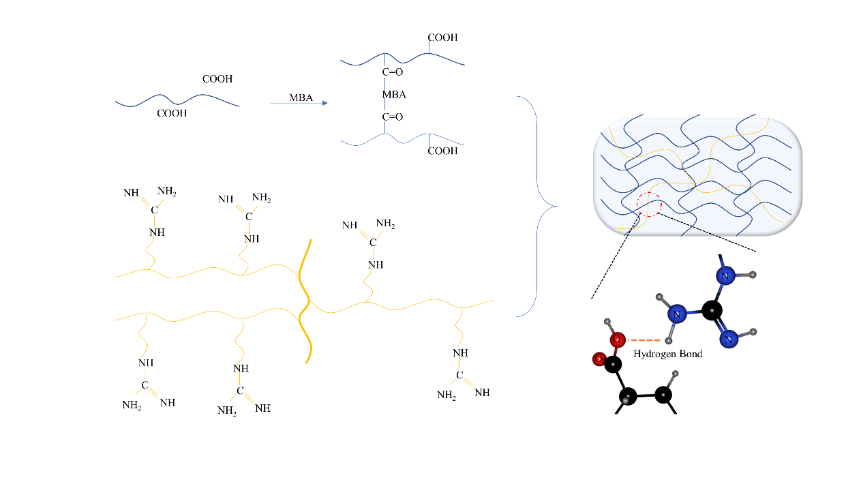


Scheme S2. A probable mechanism for synthesis of PAA/PEA hydrogel.


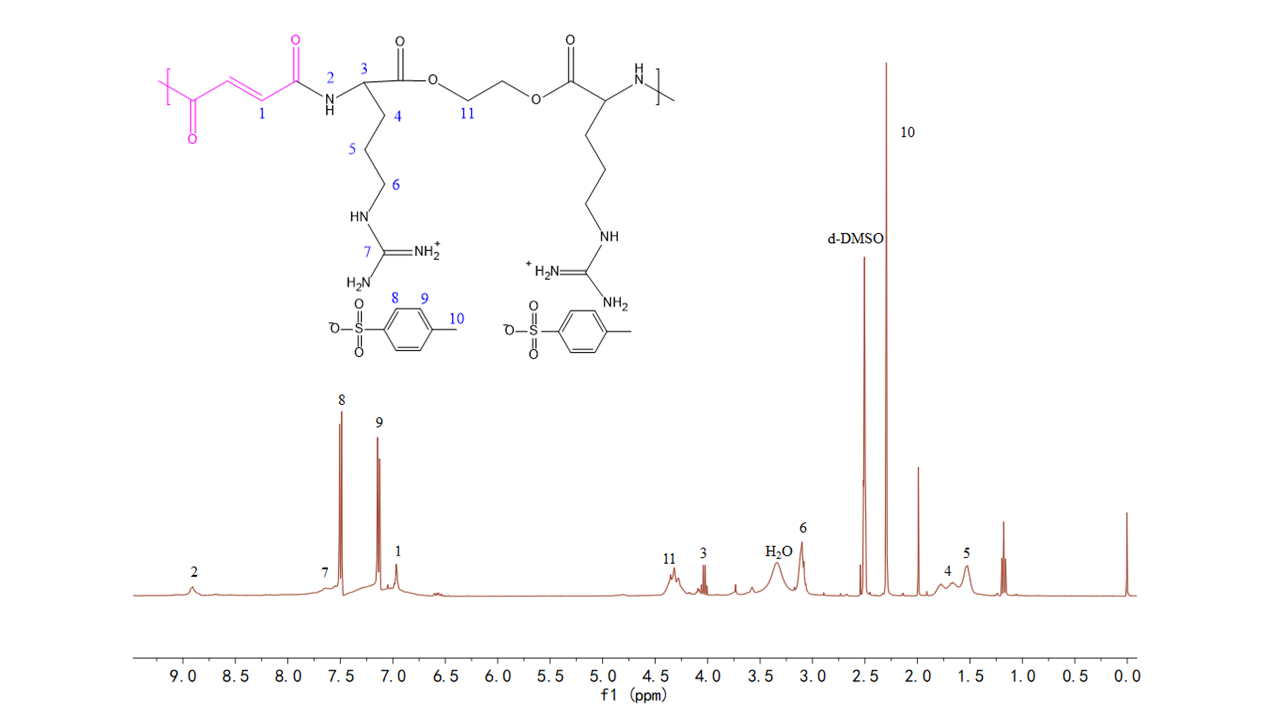


Figure S1. ^1^H NMR spectra of Arg-UPEA.


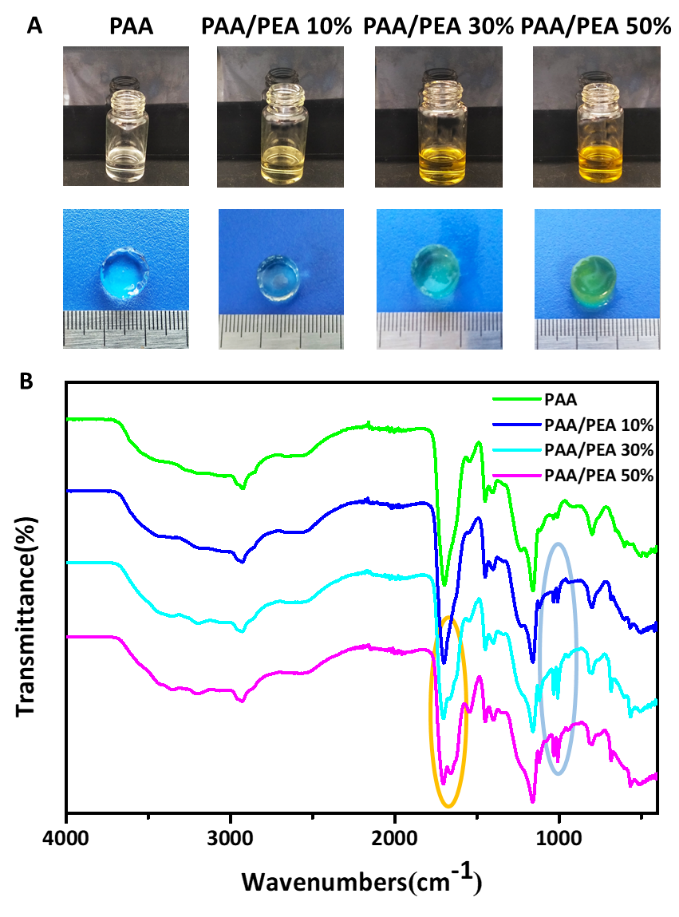


Figure S2. Macroscopic diagram of hydrogel preparation and FTIR spectrum of hydrogels.


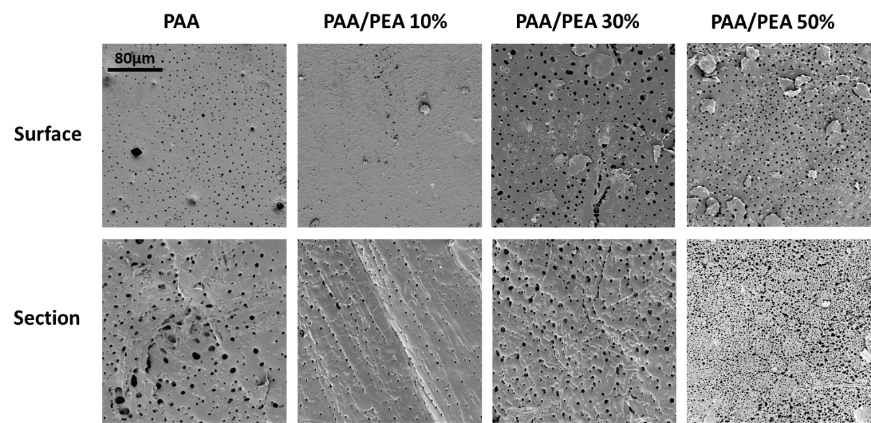
Figure S3. SEM images of hydrogels at swollen state.


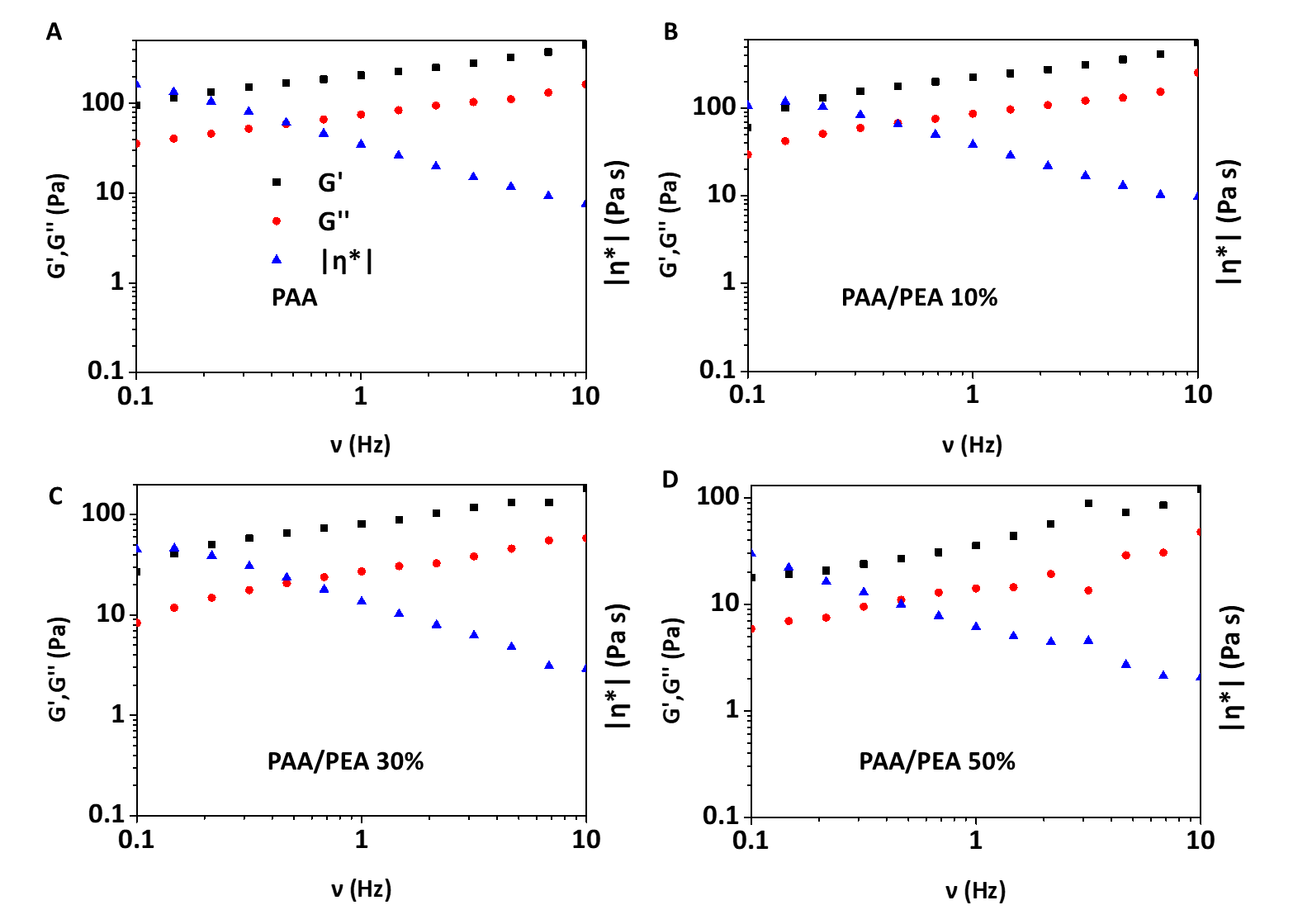
Figure S4. Rheological behavior of hydrogels.





Figure S5. DPPH scavenging percentage by hydrogels with different concentrations at 30 min.


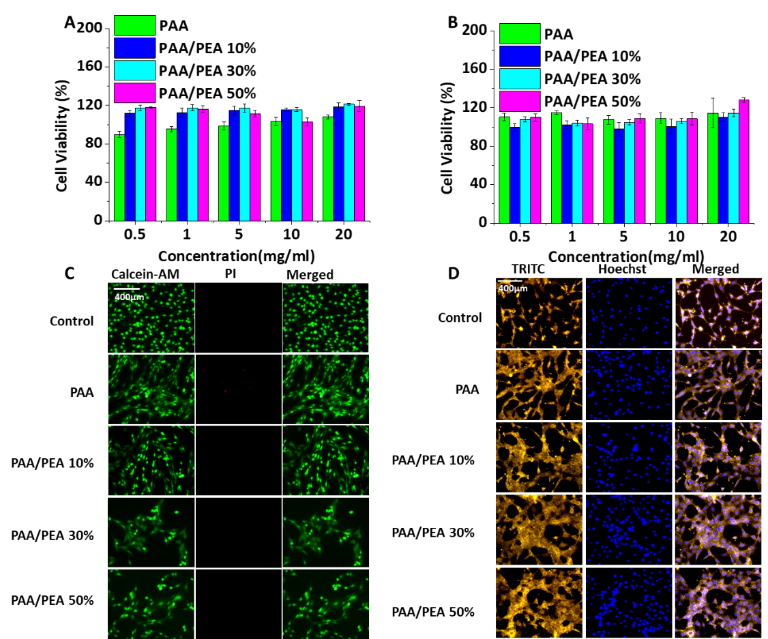


Figure S6. *In vitro* study of cells with hydrogels. MTT assay at day 3 (A) and 5 (B), and (C) live/dead staining assay to evaluate the viability of NIH 3T3 cells cultured with PAA single hydrogel or PAA/PEA hydrogels after 72 h. (D) Representative micrographs of NIH 3T3 cells after 72 h culture on the surfaces of hydrogels.


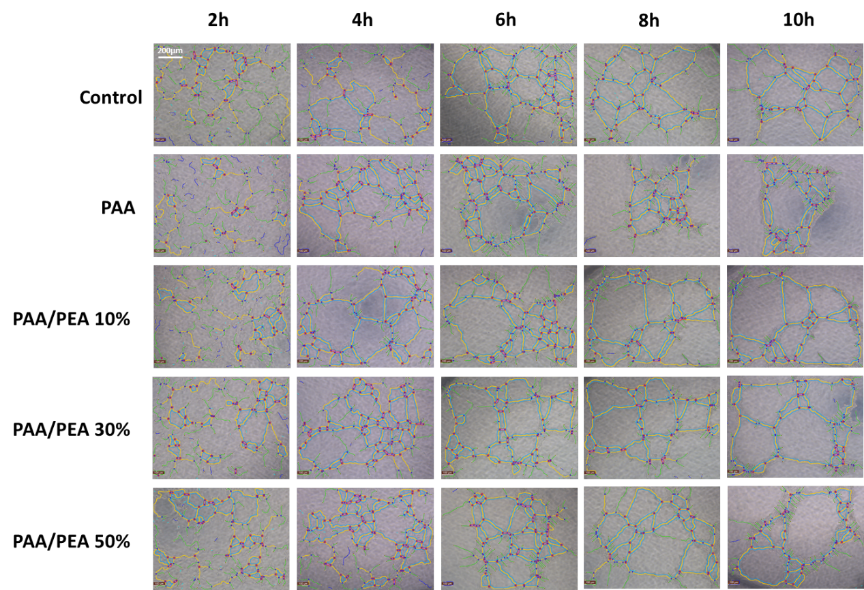
Figure S7. Hydrogels extract exposure accelerated angiogenesis in vitro. HUVECs were seeded on growth factor reduced Matrigel and incubated in serum-free medium with or without 5 mg/ml hydrogels extract. Pictures were taken at 2, 4, 6, 8 and 10 hours after seeding.


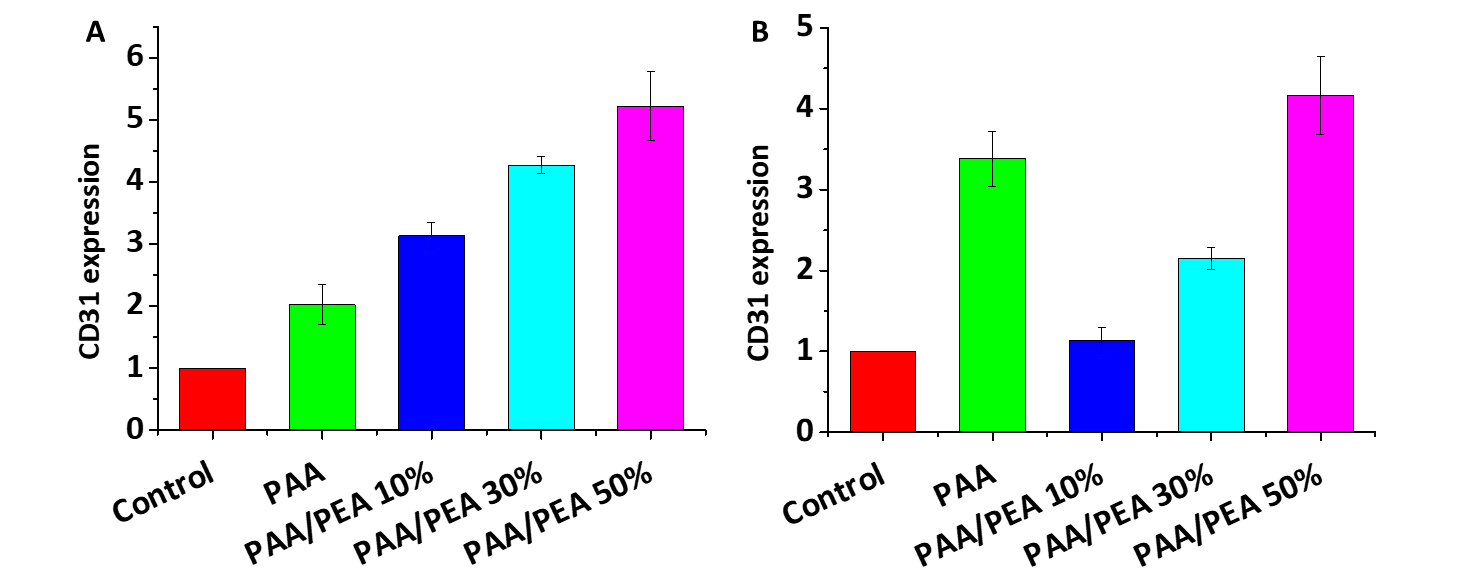
Figure S8. Quantitative analysis of CD31 using qPCR to evaluate the mRNA expression levels of neo-vascularization marker at 24 h (A) and 48 h (B).


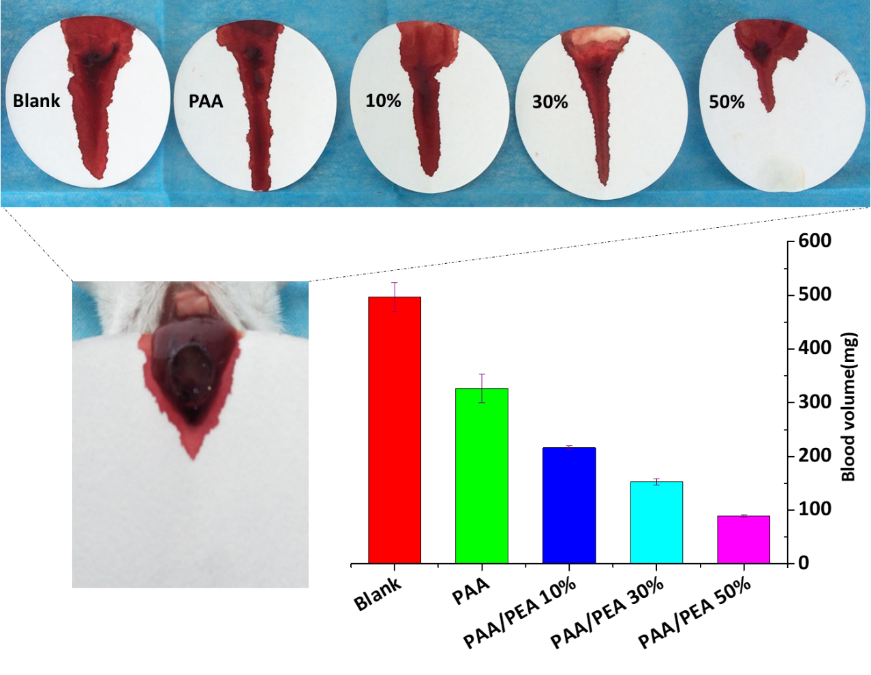
Figure S9. Hemostatic performance on rat’s liver treated with hydrogels.


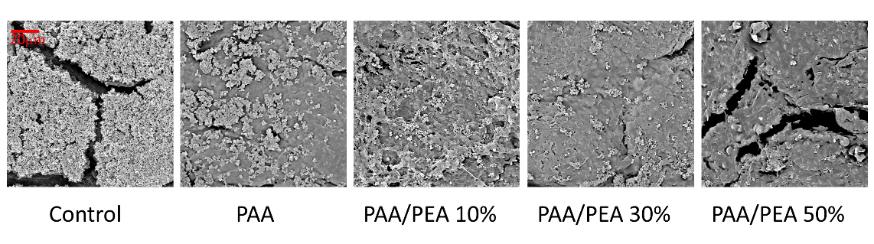


Figure S10. The antibacterial ability of PAA/PEA hybrid hydrogels.


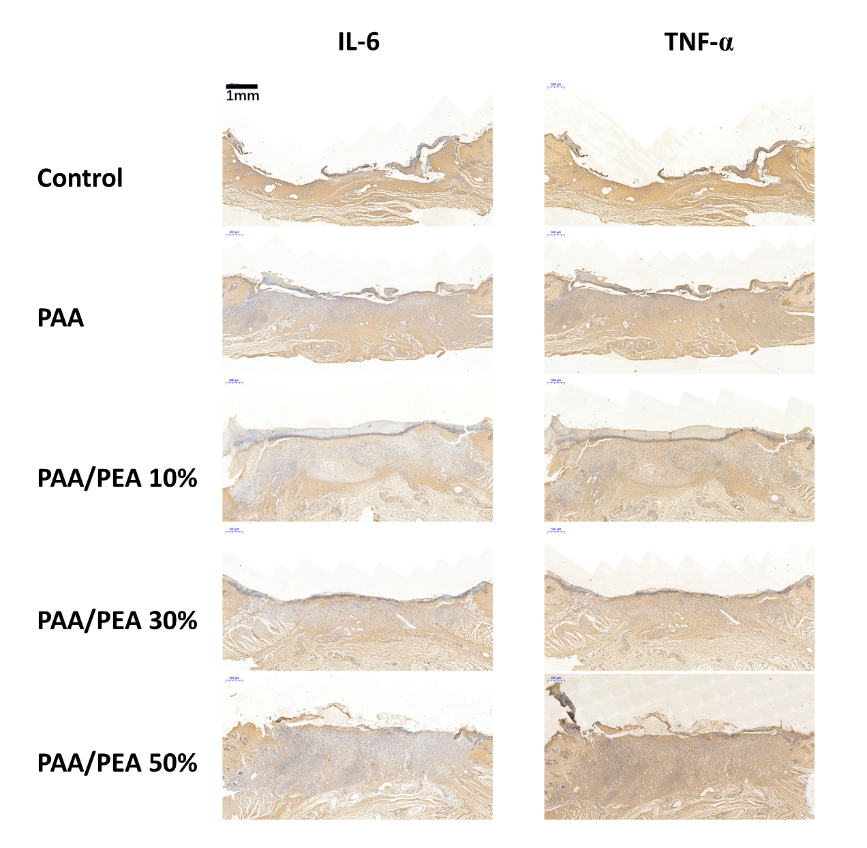


Figure S11. Representative histological IL-6 and TNF-α stained sections of wounds of rats treated on day 5.


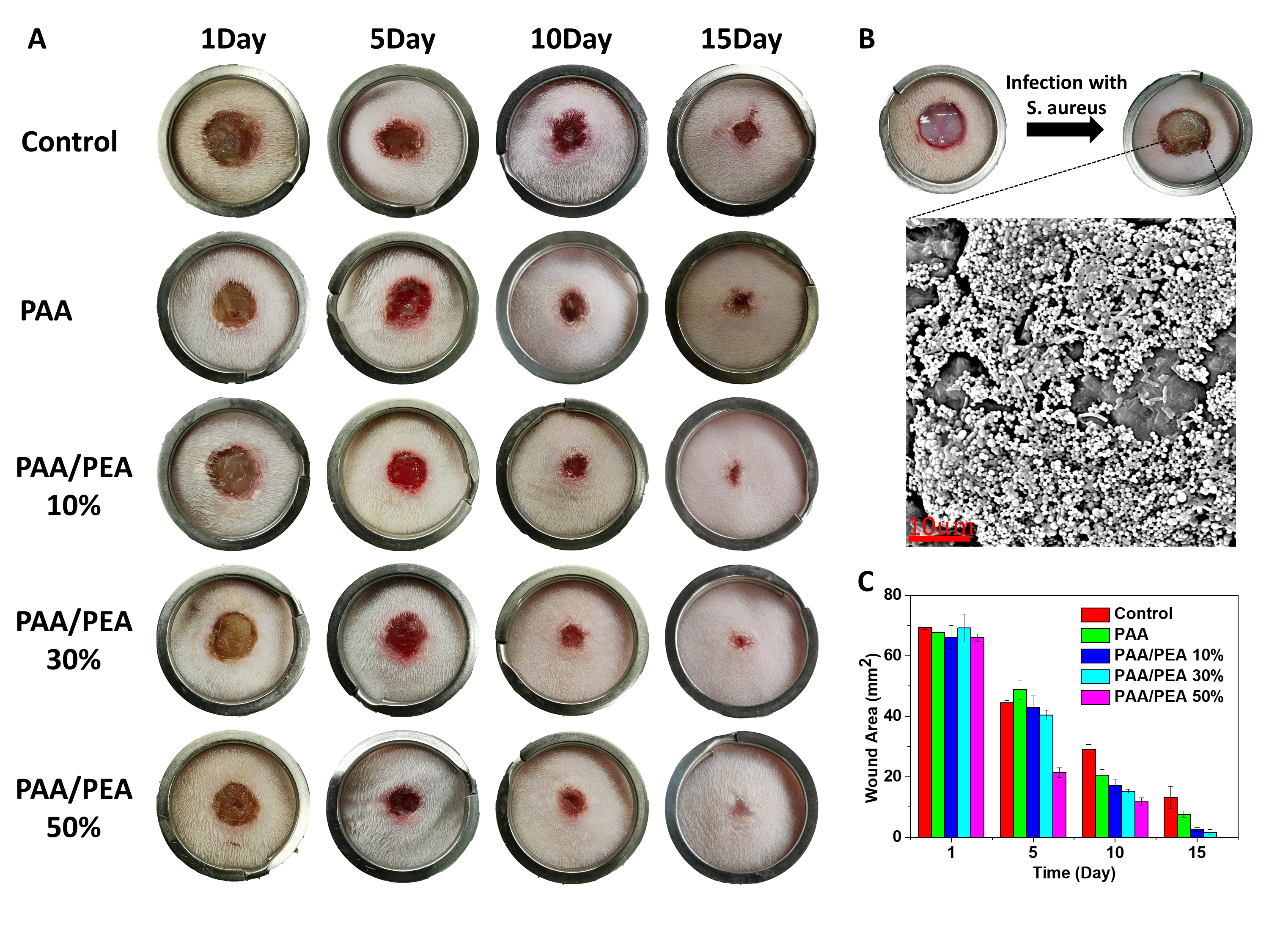


Figure S12. (A) Representative images of wounds treated with PAA, PAA/PEA10%, PAA/PEA 30%, and PAA/PEA 50% hydrogels. (B) Wound bacterial infection and the SEM image of the wound after infection. (C) Wound repair area of different experimental groups.
